# Pulmonary Procoagulant and Innate Immune Responses in Critically Ill COVID-19 Patients

**DOI:** 10.3389/fimmu.2021.664209

**Published:** 2021-05-14

**Authors:** Esther J. Nossent, Alex R. Schuurman, Tom D.Y. Reijnders, Anno Saris, Ilse Jongerius, Siebe G. Blok, Heder de Vries, JanWillem Duitman, Anton Vonk Noordegraaf, Lilian J. Meijboom, René Lutter, Leo Heunks, Harm Jan Bogaard, Tom van der Poll

**Affiliations:** ^1^ Department of Pulmonary Medicine, Amsterdam UMC, Free University Amsterdam, Amsterdam, Netherlands; ^2^ Amsterdam Cardiovascular Sciences Research Institute, Amsterdam UMC, Amsterdam, Netherlands; ^3^ Center for Experimental and Molecular Medicine, Amsterdam UMC, Academic Medical Center, University of Amsterdam, Amsterdam, Netherlands; ^4^ Amsterdam Infection & Immunity Institute, Amsterdam UMC, Amsterdam, Netherlands; ^5^ Department of Immunopathology, Sanquin Research, Amsterdam and Landsteiner Laboratory, Amsterdam UMC, University of Amsterdam, Amsterdam, Netherlands; ^6^ Emma Children’s Hospital, Department of Pediatric Immunology, Rheumatology and Infectious Diseases, Amsterdam UMC, University of Amsterdam, Amsterdam, Netherlands; ^7^ Department of Intensive Care Medicine, Amsterdam UMC, Free University Amsterdam, Amsterdam, Netherlands; ^8^ Department of Radiology and Nuclear Medicine, Amsterdam UMC, Free University Amsterdam, Amsterdam, Netherlands; ^9^ Department of Experimental Immunology, Amsterdam UMC, Academic Medical Center, University of Amsterdam, Amsterdam, Netherlands; ^10^ Department of Infectious Diseases, Amsterdam UMC, Academic Medical Center, University of Amsterdam, Amsterdam, Netherlands

**Keywords:** COVID-19, persistent ARDS, coagulation, innate immune response, bronchoalveolar space

## Abstract

**Rationale:**

Systemic activation of procoagulant and inflammatory mechanisms has been implicated in the pathogenesis of COVID-19. Knowledge of activation of these host response pathways in the lung compartment of COVID-19 patients is limited.

**Objectives:**

To evaluate local and systemic activation of coagulation and interconnected inflammatory responses in critically ill COVID-19 patients with persistent acute respiratory distress syndrome.

**Methods:**

Paired bronchoalveolar lavage fluid and plasma samples were obtained from 17 patients with COVID-19 related persistent acute respiratory distress syndrome (mechanical ventilation > 7 days) 1 and 2 weeks after start mechanical ventilation and compared with 8 healthy controls. Thirty-four host response biomarkers stratified into five functional domains (coagulation, complement system, cytokines, chemokines and growth factors) were measured.

**Measurements and Main Results:**

In all patients, all functional domains were activated, especially in the bronchoalveolar compartment, with significantly increased levels of D-dimers, thrombin-antithrombin complexes, soluble tissue factor, C1-inhibitor antigen and activity levels, tissue type plasminogen activator, plasminogen activator inhibitor type I, soluble CD40 ligand and soluble P-selectin (coagulation), next to activation of C3bc and C4bc (complement) and multiple interrelated cytokines, chemokines and growth factors. In 10 patients in whom follow-up samples were obtained between 3 and 4 weeks after start mechanical ventilation many bronchoalveolar and plasma host response biomarkers had declined.

**Conclusions:**

Critically ill, ventilated patients with COVID-19 show strong responses relating to coagulation, the complement system, cytokines, chemokines and growth factors in the bronchoalveolar compartment. These results suggest a local pulmonary rather than a systemic procoagulant and inflammatory “storm” in severe COVID-19.

## Introduction

The severe acute respiratory syndrome corona virus (SARS-CoV)-2 pandemic has had a tremendous global impact. While most SARS-CoV-2 infections are mild, up to 20% of cases result in severe Coronavirus Disease (COVID)-19, particularly in the elderly and in patients with cardiopulmonary comorbidities (1). Severe COVID-19 is associated with respiratory failure, which has been considered a form of acute respiratory distress syndrome (ARDS), although its characteristics may differ from ARDS caused by other diseases (2, 3). Another distinctive feature of COVID-19 is the frequent occurrence of venous thrombo-embolic (VTE) events, which may be related to hypercoagulability (4, 5). These particularities of COVID-19 have raised considerable interest in the interactions between coagulation and the immune response triggered by this new disease.

The innate immune system is the first to respond to infection of the airways by SARS-CoV-2 (6). The respiratory epithelium and resident leukocytes release cytokines and chemokines that trigger recruitment of other immune cells to the primary site of infection. While this response is initiated to inhibit viral replication, unrestrained activation of inflammation can result in collateral tissue damage, which has been documented in ARDS associated with other types of lung infection (7). In addition, ARDS and pneumonia can result in aberrant activation of coagulation in the lung microenvironment. While this pulmonary coagulopathy is largely inflammation-driven, coagulation proteases in turn can amplify inflammation, resulting in an injurious vicious cycle (8–10).

Previous investigations have reported exuberant activation of the innate immune and coagulation systems in the systemic circulation of patients with COVID-19 (4, 11–14). Knowledge on local activation of these host response pathways in ventilated COVID-19 is limited. Autopsy studies have shown extensive alveolar damage accompanied by widespread inflammation and pulmonary *in situ* thrombosis in patients who succumbed to COVID-19 (2, 15). Recently, it was proposed that SARS-CoV-2 infection induces a process termed immunothrombosis, in which activated leukocytes interact with platelets and coagulation factors, leading to intravascular clot formation and microthrombotic complications in lungs and other organs (16). Here we set out to evaluate local and systemic activation of coagulation and interconnected inflammatory responses in critically ill patients with COVID-19 by measuring a large set of biomarkers in bronchoalveolar lavage fluid (BALF) and concurrently collected plasma. We hypothesized that severe COVID-19 would be associated with strong activation of coagulation especially locally in the lungs and that this would be associated with concurrent activation of host response pathways implicated in coagulation and lung injury. To test this we composed a set of 34 host response biomarkers reflection alterations in five pathophysiological domains, i.e., coagulation, the complement system, cytokines, chemokines and growth factors.

## Methods

### Study Design

This study was part of the Amsterdam Study for Deep Phenotyping of COVID-19 disease (ArtDECO) 1 study, a cohort study of all patients with PCR confirmed COVID-19 related persistent ARDS (mechanical ventilation > 7 days) admitted to the intensive care unit (ICU) of the Amsterdam University Medical Centers (Amsterdam UMC), location VUmc. ARDS was defined according to the Berlin criteria (17). Per clinical protocol all patients requiring more than 7 days of mechanical ventilation underwent video-assisted bronchoscopy BALF sampling. Left-over biological samples and clinical data were stored in the anonymized research Amsterdam UMC COVID-19 biobank (#2020-182) and database (Castor; castoredc.com). Informed consent for the use of samples and data was deferred until discharge from the ICU. In case of death, informed consent was requested from the patient’s relatives. The study procedure was approved by the Review Committee of the Amsterdam UMC Biobank (protocol number 2020-065). The study was performed in accordance with the declaration of Helsinki and adheres to Dutch regulations. The current investigation included patients from whom data and samples were available between March 27th and May 31st 2020. All available paired BALF-plasma samples harvested between 1 and 2 weeks after start invasive mechanical ventilation were used for this analysis; from a subset of patients obtained paired follow up samples were also analyzed. Techniques concerning blood and BALF sampling, and used assays, are described in the detail in the online data supplement. Biological samples were compared with samples of 8 healthy subjects (5/8 male, mean age 38.75 years, 1/8 ex-smoker) from whom BALF (8 x 20 ml 0,9% NaCl) and plasma was obtained as part of study protocols NL48912.018.14 (RILCA trial) and NL53354.018.15 (RILCO trial) approved by the institutional ethics committee after having given written informed consent.

### Clinical Protocol

Per clinical protocol, all patients with PCR confirmed COVID-19 related persistent ARDS requiring more than 7 days of mechanical ventilation, admitted to the intensive care unit (ICU) of the Amsterdam University Medical Centers (Amsterdam UMC), location VUmc, underwent chest computed tomography (CT) without and with (when clinically indicated) intravenous contrast (CT pulmonary angiography (CTPA)) at fixed time-points. At the same time, respiratory mechanics were measured and video-assisted bronchoscopy BALF sampling was performed. These procedures were repeated on a weekly basis for as long as patients were intubated and did not show clinical improvement. Intravenous steroid treatment with 1mg/kg prednisone, with a maximum dose of 80 mg once a day, was started after fourteen days of mechanical ventilation, in absence of clinical improvement and after exclusion of pulmonary infectious complications. Steroids were tapered after 10 days. When clinically indicated, chest CT and bronchoscopy with BALF sampling were also performed in addition to these fixed time points.

### Blood and BALF Sampling

Prior to diagnostic BALF sampling, venous blood was drawn in EDTA anticoagulated tubes. Blood was centrifuged 10 min at 1800g and supernatant plasma was collected and stored at -80°C. During bronchoscopy lungs were instilled with 2 x 20 ml 0,9% NaCl at a (sub)segmental level, each aspirated immediately with low suction for microbiological diagnostic purposes. Leftover BALF (3-20 ml) was centrifuged (300g, 10min, 4°C) and BALF supernatant was stored at -80°C until further analysis.

### Assays

BALF was treated with 1% Triton-X100 for 2 hours before samples were used for the specific assays to eliminate all viable virus. D-dimer, soluble tissue factor, tissue type plasminogen activator (tPA), plasminogen activator inhibitor type I (PAI-1), soluble CD40 ligand (sCD40L) and soluble P-selectin (sP- selectin) were measured using Human Thrombosis LEGENDplex™ (#740892, BioLegend, Amsterdam, the Netherlands). Thrombin-antithrombin complexes (TATc) were measured by ELISA (TAT-EIA, Affinity Biologicals, Leiden, the Netherlands). Kallikrein-C1-inhibitor complexes (18) C3bc (19) C4bc (20) mannose binding lectin (MBL) (21) and C1-inhibitor (C1-INH) antigen (22) and activity (22) were measured by assays as described previously. All other mediators and growth factors were measured by Human XL Cytokine Magnetic Luminex Performance Assay (#LKTM014, R&D systems, Abingdon, United Kingdom) and were read on a Bioplex 200.

### Statistical Analysis

All results are presented as numbers (percentages) for categorical variables, median and interquartile ranges (IQR, 25th and 75th percentiles) for non-parametric quantitative variables (boxplots) and mean ± standard deviation (SD) for parametric quantitative variables. Differences between groups were tested by Wilcoxon signed-rank test for paired data and Wilcoxon rank sum test for unpaired data. A p value ≤ 0.05 was considered statistically significant. All statistical analyses were performed in R (version 4.0.2; R Foundation for Statistical Computing, Vienna, Austria).

## Results

### Patients and Analysis of the Local and Systemic Host Response

Seventeen patients from whom paired BALF and plasma samples were harvested in parallel between 1 and 2 weeks after initiation of invasive mechanical ventilation were studied. Clinical characteristics are shown in **Table 1**. Patient demographics were comparable to previous reports on COVID-19 disease with a higher incidence in elderly, male subjects with an elevated BMI (1, 11, 12, 23). In eleven patients (64.7%) treatment with steroids was initiated. Ten patients started after baseline BALF and plasma sampling and 1 patient just prior to baseline sampling. Seven patients (41.2%) were treated with hydroxychloroquine, 2 patients (11.8%) with the tyrosine kinase inhibitor imatinib. All patients underwent CTPA within 7 days of ICU admission; ten patients (58.8%) were diagnosed with pulmonary embolism, for which therapeutic anticoagulation was initiated. Thirteen patients (76.5%) were treated for a possible secondary infectious complication during their stay in the ICU (**Table 2**). Four patients died during ICU stay (23.5%).

**Table 1:** Demographics and clinical characteristics of patients with COVID-19 related persistent acute respiratory distress syndrome at ICU admission.

**Number of patients (n)**	17
**Demographics**	
Male/Female	17/0
Age (years), mean (standard deviation)	63.4 (10.6)
Body mass index (m^2^/kg), mean (standard deviation) (n=16)	29.7 (4.8)
**Medical history**	
No significant comorbidities	6 (35.3%)
Diabetes	5 (29.4%)
Chronic obstructive pulmonary disease/asthma	2 (11.8%)
Cardiovascular disease	6 (35.3%)
Active malignancy	1 (5.9%)
Human immunodeficiency virus infection	1 (5.9%)
**Severity of disease**	
Sequential organ failure assessment score total, median (interquartile range) (n=11)	9 (3)

**Table 2:** Secondary pulmonary infections during ICU stay.

	Patients
Bacterial superinfection	3
Probable invasive pulmonary aspergillosis	1
Herpes simplex virus reactivation	4
Bacterial superinfection and probable invasive pulmonary aspergillosis	1
Bacterial superinfection and Herpes simplex virus reactivation	1
Cytomegalovirus reactivation	3

Table lists infections for which the clinical team started specific therapy.

Activation of procoagulant and immune responses in the bronchoalveolar compartment of critically ill COVID-19 patients were measured using a comprehensive set of biomarkers, stratified into five functional “domains”, i.e., coagulation, the complement system, cytokines, chemokines and growth factors. To obtain insight into the extent of compartmentalization of these responses, the same biomarkers were measured in plasma.

### Coagulation Activation

**Figure 1:**
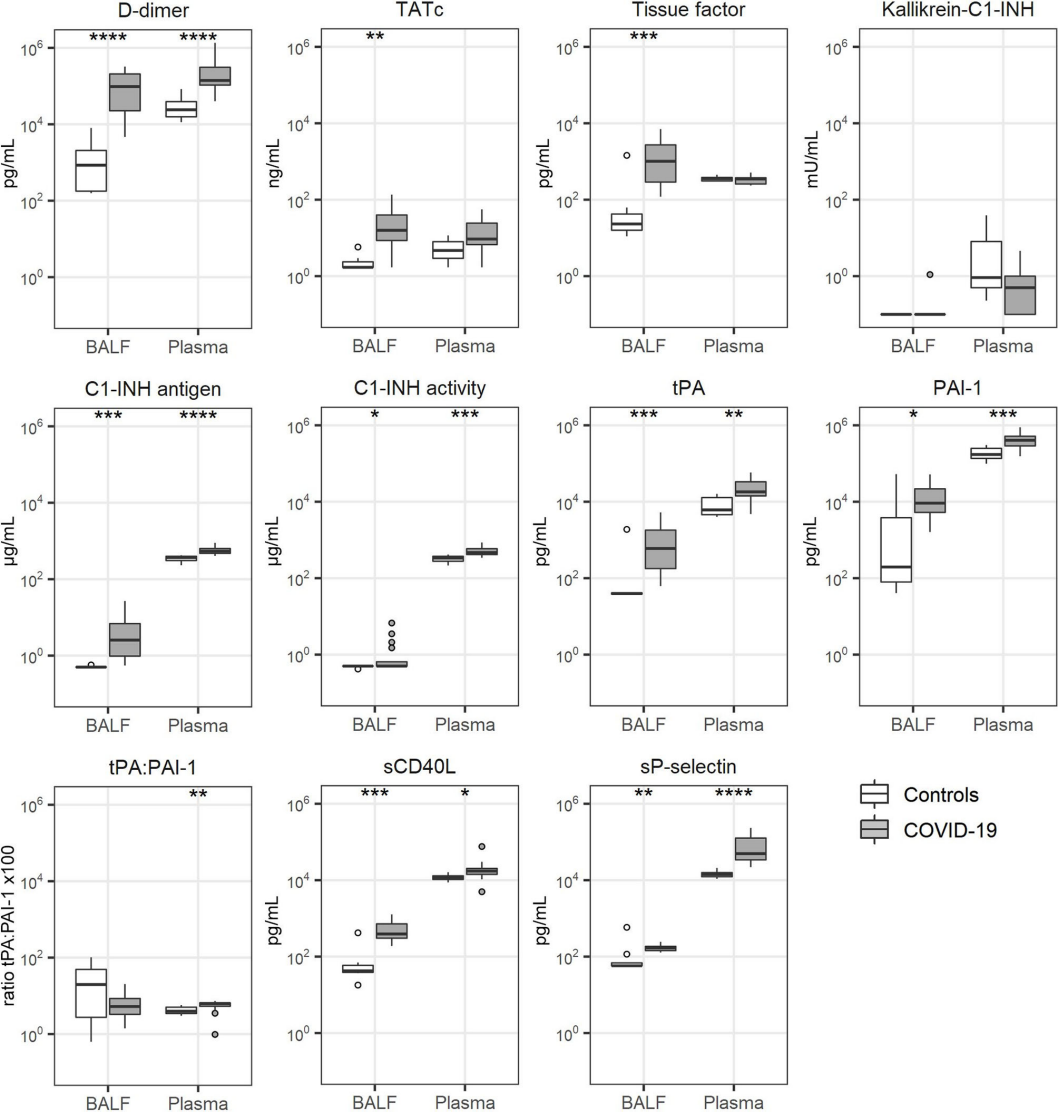
Coagulation activation. Bronchoalveolar lavage fluid and plasma were obtained from 17 critically ill COVID-19 patients who had been on mechanical ventilation for at least 7 days and 8 healthy control subjects. Data are expressed as box and whisker diagrams depicting the median and lower quartile, upper quartile, and their respective 1.5 interquartile range as whiskers (as specified by Tukey). Comparisons between groups were performed using the Wilcoxon rank sum test. *p ≤ 0.05, **p ≤ 0.01, ***p ≤ 0.001, ****p = ≤ 0.0001. BALF, bronchoalveolar lavage fluid; C1-INH, C1-inhibitor; Kallikrein-C1-INH, Kallikrein-C1-inhibitor complexes; PAI-1, plasminogen activator inhibitor type I; sCD40L, soluble CD40 Ligand; sP-selectin, soluble P-selectin; TATc, thrombin-antithrombin complexes; tPA, tissue type plasminogen activator.

Markers of coagulation activation (D-dimer, thrombin-antithrombin complexes (TATc) and soluble tissue factor) (9, 10) were strongly elevated in BALF of COVID-19 patients relative to levels measured in BALF from control subjects (**Figure 1**). Of interest, in plasma only D-dimer concentrations were elevated in patients, whereas the plasma levels of TATc and soluble tissue factor were not different from those in healthy controls. Kallikrein-C1-inhibitor complexes, reflecting activation of the contact system (24), was not detectable in BALF from either patients or controls, while plasma levels were not different between groups. C1-inhibitor (C1-INH) antigen and activity levels were elevated in both BALF and plasma from patients. The fibrinolysis markers tissue type plasminogen activator (tPA) and plasminogen activator inhibitor type I (PAI-1 (9) were markedly elevated in BALF and modestly in plasma of patients, when compared to heathy controls. tPA/PAI-1 ratio’s in BALF were not different between patients and controls, whilst in plasma these ratio’s were slightly higher in patients. Soluble CD40 ligand (sCD40L) and soluble P-selectin (sP-selectin), indicative of platelet activation (25), were increased in both BALF and plasma from patients when compared with controls.

### Complement Activation

**Figure 2:**
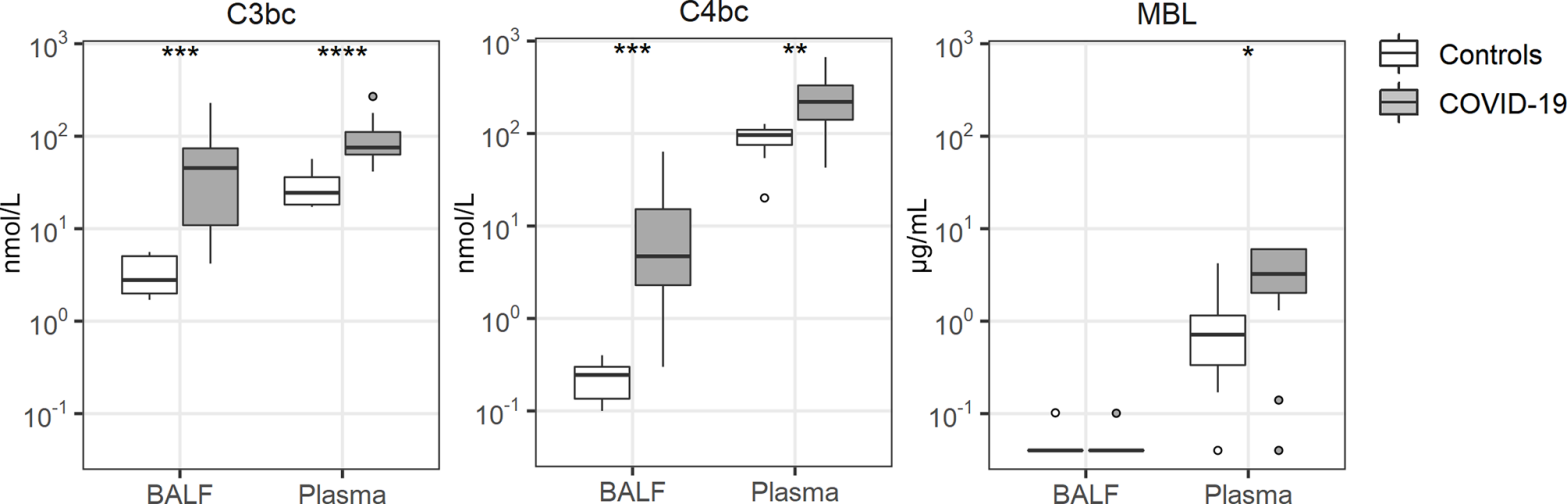
Complement activation. Bronchoalveolar lavage fluid and plasma were obtained from 17 critically ill COVID-19 patients who had been on mechanical ventilation for at least 7 days and 8 healthy control subjects. Data are expressed as box and whisker diagrams depicting the median and lower quartile, upper quartile, and their respective 1.5 interquartile range as whiskers (as specified by Tukey). Comparisons between groups were performed using the Wilcoxon rank sum test. *p ≤ 0.05, **p ≤ 0.01, ***p ≤ 0.001, ****p ≤ 0.0001. BALF, bronchoalveolar lavage fluid; C3bc, complement 3bc; C4bc, complement 4bc; MBL, mannose binding lectin.

Tight interactions exist between coagulation and the complement system (26). BALF and plasma levels of C3bc (indicative of activation of the common pathway of complement) (27) and C4bc (indicative of activation of the classical and lectin pathways of complement) (27) were strongly increased in COVID-19 patients relative to controls (**Figure 2**). In contrast, mannose binding lectin (MBL) levels remained undetectable in BALF, while plasma MBL was higher in patients than in controls.

### Cytokine and Chemokine Release

**Figure 3:**
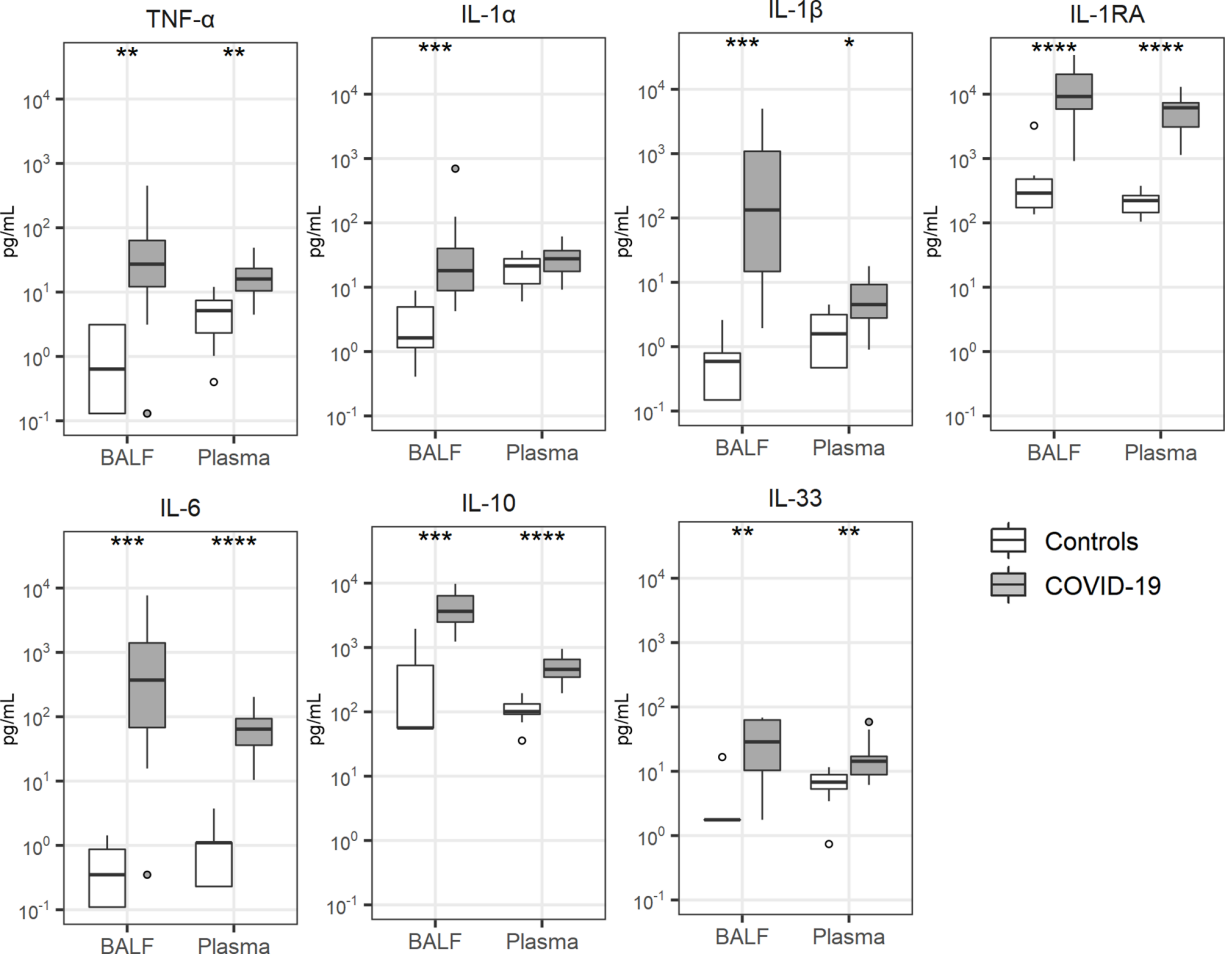
Cytokine release. Bronchoalveolar lavage fluid and plasma were obtained from 17 critically ill COVID-19 patients who had been on mechanical ventilation for at least 7 days and 8 healthy control subjects. Data are expressed as box and whisker diagrams depicting the median and lower quartile, upper quartile, and their respective 1.5 interquartile range as whiskers (as specified by Tukey). Comparisons between groups were performed using the Wilcoxon rank sum test. *p ≤ 0.05, **p ≤ 0.01, ***p ≤ 0.001, ****p ≤ 0.0001. BALF, bronchoalveolar lavage fluid; IL, interleukin; IL-1RA, interleukin-1 receptor antagonist; TNF-α, tumor necrosis factor-α.

**Figure 4:**
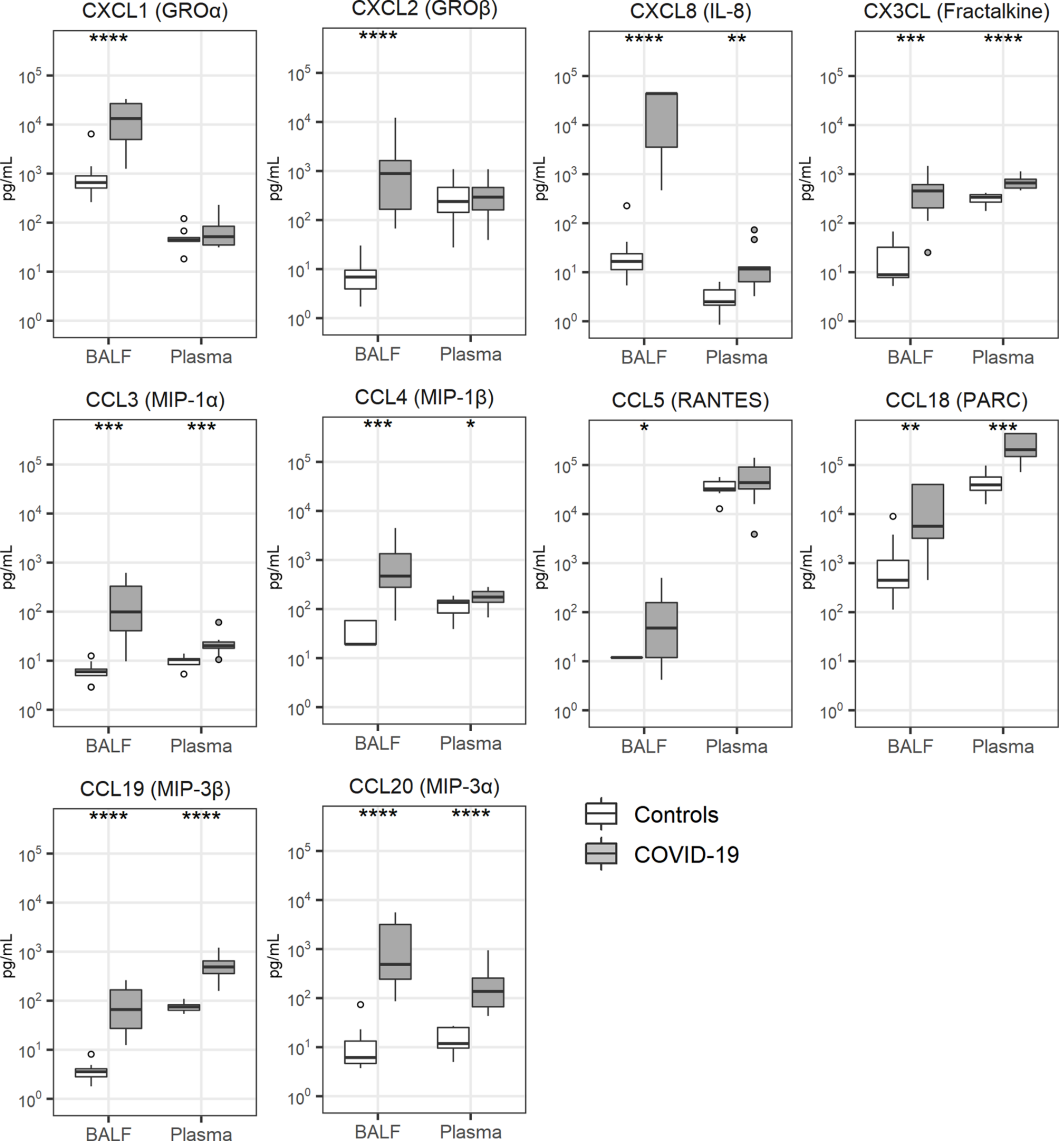
Chemokine release. Bronchoalveolar lavage fluid and plasma were obtained from 17 critically ill COVID-19 patients who had been on mechanical ventilation for at least 7 days and 8 healthy control subjects. Data are expressed as box and whisker diagrams depicting the median and lower quartile, upper quartile, and their respective 1.5 interquartile range as whiskers (as specified by Tukey). Comparisons between groups were performed using the Wilcoxon rank sum test. *p ≤ 0.05, **p ≤ 0.01, ***p ≤ 0.001, ****p ≤ 0.0001. BALF, bronchoalveolar lavage fluid; IL, interleukin; MIP, macrophage inflammatory protein; PARC, pulmonary and activation-regulated chemokine; RANTES, Regulated upon Activation, Normal T Cell Expressed and Presumably Secreted.

Local cytokine release has been implicated in the pathogenesis of lung injury and pulmonary coagulopathy in patients with ARDS (7, 9) and several investigations reported a “cytokine storm” in patients with COVID-19, referring to elevated plasma concentrations of cytokines and chemokines (28, 29). Concentrations of cytokines were particularly elevated in BALF of patients with COVID-19 (**Figure 3**). This was true for proinflammatory cytokines (tumor necrosis factor (TNF)-α, interleukin (IL)-1α, IL-1β), anti-inflammatory cytokines (IL-1 receptor antagonist) as well as cytokines with a mixed functional profile (IL-6, IL-10, IL-33). The plasma levels of these cytokines were also higher in patients than controls. Likewise, all 10 chemokines measured, were elevated in BALF of patients when compared with that from controls; in plasma chemokine levels were more modestly (CXCL8, CX3CL, CCL3, CCL4, CCL18, CCL19, CCL20) or not elevated (CXCL1, CXCL2, CCL5) in patients (**Figure 4**).

### Growth Factor Release

**Figure 5:**
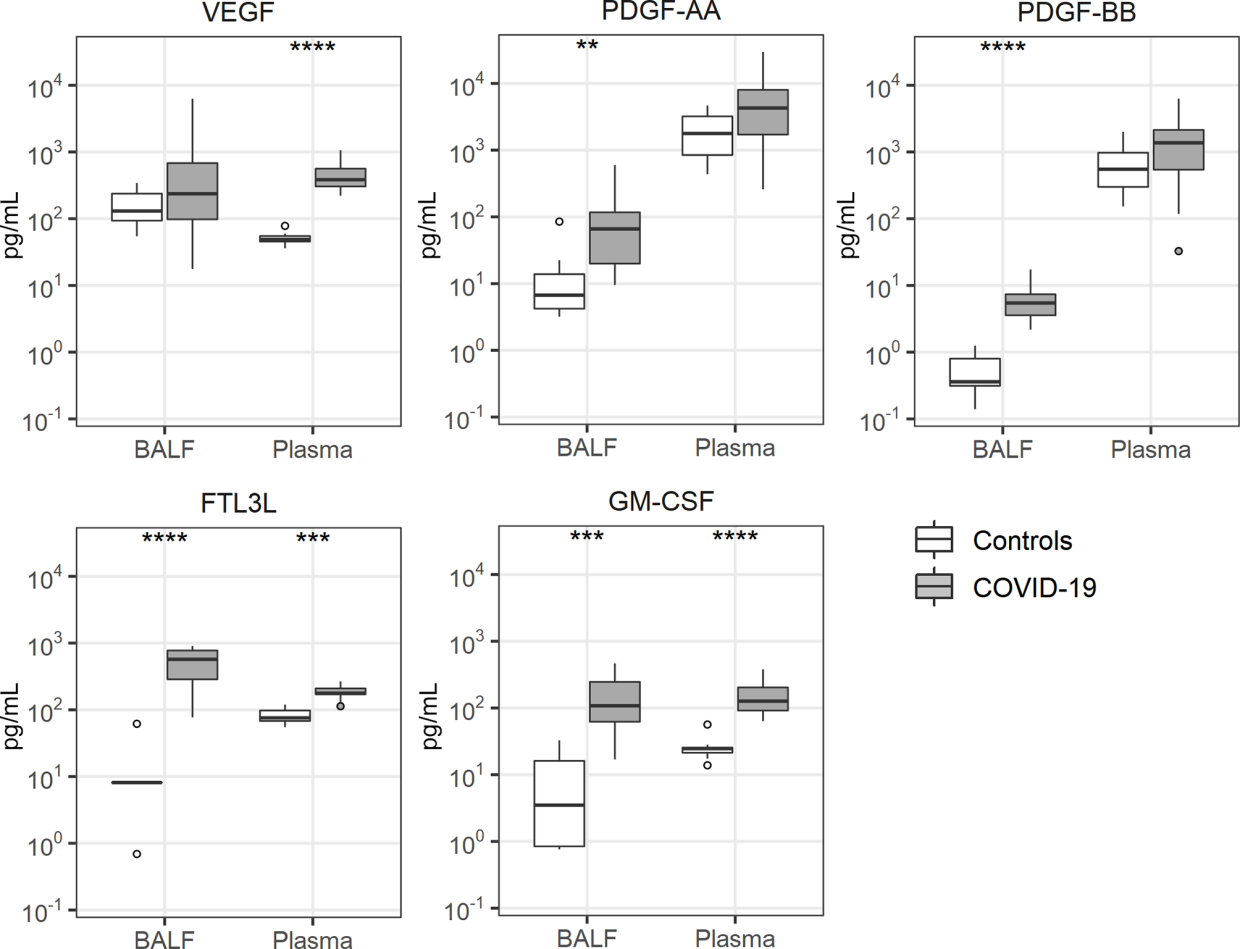
Growth factor release. Bronchoalveolar lavage fluid and plasma were obtained from 17 critically ill COVID-19 patients who had been on mechanical ventilation for at least 7 days and 8 healthy control subjects. Data are expressed as box and whisker diagrams depicting the median and lower quartile, upper quartile, and their respective 1.5 interquartile range as whiskers (as specified by Tukey). Comparisons between groups were performed using the Wilcoxon rank sum test. **p ≤ 0.01, ***p ≤ 0.001, ****p ≤ 0.0001. BALF, bronchoalveolar lavage fluid; FTL3L, fms like tyrosine kinase 3 ligand; GM-CSF, granulocyte-macrophage colony-stimulating factor; PDGF, platelet derived growth factor; VEGF, vascular endothelial growth factor.

Several growth factors have been studied in the context of lung inflammation, including vascular endothelial growth factor (VEGF) (30), platelet derived growth factor (PDGF)-AA (31), PDGF-BB (31), fms like tyrosine kinase 3 ligand (FLT3L) (32) and granulocyte-macrophage colony stimulating factor (GM-CSF) (33). Of these, PDGF-AA and PDGF-BB were only elevated in BALF of patients with COVID-19, while FLTL3 and GM-CSF were elevated in BALF more so than in plasma (**Figure 5**). VEGF was only increased in plasma of patients.

### Temporal Changes in Local and Systemic Procoagulant and Immune Responses During ICU Stay

From ten patients follow up BALF and plasma samples were obtained 3 or 4 weeks after start invasive mechanical ventilation. Across all five functional domains many host response biomarker levels in BALF showed decreasing trends between week 1-2 and week 3-4, reaching statistical significance for soluble tissue factor, tPA, sCD40L, sP-selectin (coagulation, **Supplementary Figure 1**), TNF- α, IL-1α, IL-1β (cytokine release, **Supplementary Figure 3**), CXCL2, CXCL8, CCL3, CCL4 (chemokine release, **Supplementary Figure 4**), VEGF, PDGF-AA and FTL3L (growth factor release, **Supplementary Figure 5**). Complement activation products did not change over time in BALF (**Supplementary Figure 2**). In plasma, significant decreases were detected between week 1-2 and week 3-4 for TATc, PAI-1, IL-1 receptor antagonist, IL-6, IL-10, CXCL8, CCL19, CCL20, VEGF and GM-CSF (**Supplementary Figures 6–10**). There was no clear relationship between treatment with corticosteroids and changes in biomarker levels between 1-2 and 3-4 weeks (see color codes for absence or presence of corticosteroid treatment in individual patients in **Supplementary Figures 1–10**).

### Differences Between Patients With and Without Pulmonary Embolism

Comparison of host response biomarkers between patients with pulmonary embolism (n = 10) and those without pulmonary embolism (n =7) revealed higher IL-6 plasma levels and in BALF lower C1-inhibitor activity, lower IL-10 and lower GM-CSF in patients with pulmonary embolism; **Supplementary Table 1**).

## Discussion

Here we report an in depth biomarker analysis, both of the bronchoalveolar and systemic compartment, in consecutive ventilated critically ill COVID-19 patients admitted to the ICU who had needed mechanical ventilation for at least 7 days. Since the first description of COVID-19, many studies have documented activation of procoagulant and inflammatory pathways in the systemic circulation (4, 11, 14). We studied local activation of the coagulation system and interconnected inflammatory networks in the bronchoalveolar compartment during ICU stay. By measuring thirty-four host response biomarkers in paired BALF and plasma samples we demonstrate an especially strong response in the bronchoalveolar compartment of COVID-19 patients across five functional domains, i.e., coagulation, complement system, cytokines, chemokines and growth factors.

We show strong activation of coagulation in the respiratory system of COVID-19 patients, as reflected by elevated BALF levels of D-dimer and TATc, and in addition provide indirect evidence for a role for tissue factor herein, as suggested by highly elevated soluble tissue factor concentrations in BALF. Differences in these coagulation markers between patients and controls were much greater in BALF than in plasma, suggesting local activation of coagulation. SARS-CoV-2 may in part promote pulmonary coagulopathy by a direct effect on bronchial epithelial cells *via* activation of tissue factor signaling and impairment of epithelial anticoagulant mechanism (34). Notably, stronger activation of coagulation locally versus systemically in our ICU/ARDS cohort does not preclude a role for systemic coagulation activation in various thromboembolic and vascular events in COVID-19 in general (35). tPA/PAI-1 ratio’s were not different between groups in BALF and only modestly elevated in plasma of patients, arguing against a strongly disturbed fibrinolyic balance in COVID-19 patients and suggesting that elevated D-dimer levels reflect enhanced coagulation rather than hyperfibrinolysis. Besides *via* tissue factor-Factor VIIa, the coagulation system can be activated *via* the intrinsic pathway, which is tightly connected with the kallikrein-kinin system. Dysregulation of the kinin pathway has been suggested to contribute to pulmonary edema in COVID-19 and interventions inhibiting bradykinin activity or formation have been proposed as a potential therapy for COVID-19 (36). Activation of the kallikrein-kinin system *in vivo* is difficult to measure due to fast clearance of its proteases and protease-C1-inhibitor complexes (22). Thus, our results do not exclude activation of the kallikrein-kinin system, although one might argue that sufficient inhibitory capacity remained available in BALF and plasma, as indicated by elevated C1-inhibitor activity levels in COVID-19 patients.

Aberrant activation of the complement system has been implicated in COVID-19 coagulopathy and associated lung injury (37–41). We here provide evidence for not only activation of the complement system in the circulation, but also in the bronchoalveolar lumen of patients with COVID-19, as reflected by elevated C3bc and C4bc levels in BALF. In agreement, a recent study reported elevated C5a-desarg levels in BALF of four patients with ARDS due to COVID-19 (37). COVID-19 patients had elevated plasma MBL concentrations, suggesting involvement of the lectin pathway; in BALF MBL remained undetectable in both patients and controls. Of note, undetectable MBL levels in BALF of COVID-19 patients in the presence of high plasma MBL concentrations could reflect local activation of MBL, considering that this takes place at cell surfaces. Monocyte-derived macrophages from BALF of COVID-19 patients showed increased ficolin-1 mRNA expression, which may support local activation of the MBL pathway (42). Thus, the main route of complement activation in the lungs of COVID-19 patients remains to be determined. Besides complement products, proinflammatory cytokines such as TNF-α, IL-1α, IL-1β and IL-6 can activate coagulation, primarily *via* enhancing tissue factor expression (9, 10). We here report elevated concentrations of many proinflammatory cytokines, particularly in BALF, in COVID-19 patients

VEGF is a pluripotent glycoprotein that is constitutively expressed at high levels in the lung, where it may facilitate repair mechanisms following injury by epithelial regeneration (30). We found strongly elevated VEGF plasma levels in COVID-19 patients, while VEGF concentrations in BALF were highly variable and statistically not different from control values. VEGF may reduce vascular barrier function in the lungs and it has been postulated that angiotensin-converting enzyme (ACE)2 can antagonize this VEGF effect (43). Since SARS-CoV-2 cell invasion lowers expression of ACE2 (44), a possible detrimental role of VEGF vascular permeability could be enhanced during COVID-19; clearly, this hypothesis needs conformation in experimental settings. Elevated PDGF levels in BALF of COVID-19 patients may reflect activation of multiple cell types, including platelets, mast cells and the epithelium (31). Of interest, thrombin can induce PDGF release by human lung epithelial cells (45), pointing at a possible interaction between coagulation and PDGF production in the airways. Thus far, PDGF expression in ARDS or pneumonia has not been studied and its role in acute lung inflammatory conditions is speculative. Experimental studies have indicated that FLT3L may exert strong effects in the lung compartment. Pretreatment of mice with FLT3L increased lung injury during pneumococcal pneumonia, likely through inducing accumulation of proinflammatory dendritic cells (32), and pharmacological inhibition of FLT3 signaling attenuated LPS-induced lung injury and edema in mice (46). These studies suggest that the strongly elevated FLT3 levels in BALF of COVID-19 patients may contribute to lung injury.

Current knowledge of local activation of inflammatory mechanisms in the airways of patients with COVID-19 is limited. In severe COVID-19 lung macrophages displayed high expression of IL-1β, IL-6, TNF-α and various chemokines (CCL2, CCL3, CCL4, CCL7, CXCL9, CXCL10 and CXCL11). These patients had high IL-1β, IL-6 and IL-8 protein levels in BALF (42), a finding that was reproduced in a study entailing four patients with COVID-19 associated ARDS (37). We here expand these earlier reports to protein level and to procoagulant and inflammatory systems implicated in lung injury. We did not find evidence for a systemic “cytokine storm” in COVID-19 as plasma cytokine levels did not exceed 100 pg/mL. In line with previous reports (47, 48) our results show higher cytokine levels in the bronchoalveolar compartment suggesting an ongoing local hyperinflammatory state in severe COVID-19 patients rather than a systemic response. Our investigation adds information about a large set of biomarkers across five functional domains to these earlier studies (47, 48).

Our study has limitations. The first samples were obtained after at least 7 days on the ICU; admission samples would have provided insight into early activation of proinflammatory and procoagulant pathways during severe COVID-19. The sample size was relatively small although still considerably larger than previous studies evaluating host responses in the bronchoalveolar space of COVID-19 patients (37, 42, 47, 48). In accordance with previous studies (1, 49) we documented pulmonary emboli in 58.8% of the patients included in our investigation. It has been suggested that procoagulant and inflammatory responses (“thrombo-inflammation”) are involved herein (16, 50). Whilst we found limited differences in plasma and BALF biomarkers between patients with and without pulmonary embolism, our investigation was not designed or powered to detect such differences. Despite the selection of a specific population of COVID-19 patients there was still unavoidable heterogeneity within this observational cohort that may have affected biomarker levels, including steroid treatment in 64.7% of patients which per clinical protocol was started after two weeks of ICU stay and thus could have modified host response parameters in follow up samples obtained after 3-4 weeks. Finally, we used samples from healthy subjects who were not matched with regard to demographics, smoking or comorbidities as reference; our investigation did not entail critically ill patients without COVID-19, thereby precluding conclusions on the disease-specificity of the host response aberrations reported.

In conclusion, we report a strong local response in the lung compartment across multiple functional domains related to coagulation and inflammation in patients with persistent ARDS due to COVID-19. Early in the pandemic many studies suggested an important role for a systemic “cytokine storm” in the pathophysiology of severe COVID-19 (14, 28, 29). The current results suggest a local rather than a systemic procoagulant and inflammatory “storm” in these patients.

## Data Availability Statement

The original contributions presented in the study are included in the article/**Supplementary Material**. Further inquiries can be directed to the corresponding author.

## Ethics Statement

The studies involving human participants were reviewed and approved by Review Committee of the Amsterdam UMC Biobank (protocol number 2020-065). The patients/participants provided their written informed consent to participate in this study.

## Author Contributions

EN, JD, LM, LH, HB, and TP contributed to conception and design of the present study. EN, ARS, TR, AS, IJ, SB, HV, JD, LM, RL, LH, HB, and TP contributed to the acquisition of data. EN, ARS, TR, AS, IJ, SB, HV, JD, AV, LM, RL, LH, HB, and TP contributed to analysis or interpretation of the data. EN, ARS, and TP drafted the manuscript and TR, AS, IJ, SB, HV, JD, AV, LM, RL, LH, and HB revised the article critically for intellectual content. All authors contributed to the article and approved the submitted version.

## Funding

TR and AS are supported by the research program NACTAR (Novel Antibiotic Compounds and Therapies Antagonizing Resistance) and project multidrug resistant-phage (grant number 16447), which is financed by the Dutch Research Council (Nederlandse Organisatie voor Wetenschappelijk Onderzoek [NWO]). JD is supported by a personal grant of the Netherlands Organisation for Scientific Research (VENI grant 016.186.046). HV is supported by a research grant from Amsterdam Cardiovascular Sciences Research Institute.

## Conflict of Interest

The authors declare that the research was conducted in the absence of any commercial or financial relationships that could be construed as a potential conflict of interest.
