# Supplementary material for: Pulmonary Procoagulant and Innate Immune Responses in Critically Ill COVID-19 Patients
Source: Front Immunol. 2021 May 14;12:664209. doi: 10.3389/fimmu.2021.664209 (PMC8160522; doi:10.3389/fimmu.2021.664209)
Supplement: Supplementary file 1 [file DataSheet_1.docx]

**Online data supplement**

**Pulmonary procoagulant and innate immune responses in critically ill COVID-19 patients.**

Esther J. Nossent, MD^1,2^, Alex R. Schuurman, MD^3,4^, Tom D.Y. Reijnders, MD^3,4^, Anno Saris, PhD^3,4^, Ilse Jongerius, PhD^5,6^, Siebe G. Blok, MSc^1^, Heder de Vries, MD^2,7^, Jan Willem Duitman, PhD^3,4^, Anton Vonk Noordegraaf, MD, PhD^1,2^, Lilian J. Meijboom, MD, PhD^2,8^, René Lutter, PhD^1,4,9^, Leo Heunks, MD, PhD^2,7^, Harm Jan Bogaard, MD, PhD^1,2^, Tom van der Poll, MD, PhD^3,4,10^, on behalf of the ArtDECO consortium and the Amsterdam UMC COVID study group.

**Supplementary Table 1: Host reponse biomarkers (all pg/mL unless indicated differently) in plasma and bronchoalaveolar lavage fluid obtained between 1-2 weeks after ICU admission from patients with COVID-19 related persistent acute respiratory distress syndrome with and without pulmonary embolism**

|  | **Plasma** | | **Bronchoalveolar lavage fluid** | |
| --- | --- | --- | --- | --- |
|  | **Pulmonary embolism** | **No pulmonary embolism** | **Pulmonary embolism** | **No pulmonary embolism** |
| **Number of patients (n)** | **10** | **7** | **10** | **7** |
| **Coagulation activation** |  | | | |
| D-dimer | 15392.9  [11053.5, 48420.7] | 14017.9  [7842.9, 17684.5] | 7262.8  [2510.8, 18652.8] | 11644.0  [5981.9, 23590.4] |
| TATc (ng/mL) | 8.4  [5.7, 21.0] | 11.0  [7.0, 34.7] | 10.3  [6.3, 17.7] | 39.7  [15.5, 45.0] |
| Soluble Tissue factor | 282.4  [247.4, 353.4] | 363.0  [328.1, 387.9] | 887.1  [468.8, 2504.0] | 1012.6  [262.7, 2277.4] |
| Kallikrein-C1-INH (mU/mL) | 0.8  [0.3, 1.3] | 0.1  [0.1, 0.7] | 0.1  [0.1, 0.1] | 0.1  [0.1, 0.1] |
| C1-INH antigen (µg/mL) | 512.5  [441.0, 550.8] | 554.0  [522.5, 716.0] | 2.5  [0.6, 6.8] | 2.6  [1.7, 6.1] |
| C1-INH activity (µg/mL) | 505.0  [369.8, 584.0] | 469.0  [440.0, 656.0] | 0.5  [0.5, 0.5] | 1.5**  [0.6, 2.8] |
| tPA | 1911.8  [1674.8, 2897.0] | 1512.9  [1252.7, 4078.4] | 58.0  [9.6, 195.7] | 59.8  [29.4, 95.5] |
| PAI-1 | 39166.0  [31308.6, 47679.1] | 40482.3  [27266.9, 57674.7] | 1130.0  [385.5, 2112.5] | 918.5  [686.2, 2134.1] |
| sCD40L | 3596.2  [3117.6, 3999.2] | 3122.7  [2172.2, 4047.1] | 109.7  [72.3, 154.7] | 78.1  [55.4, 101.2] |
| sP-selectin | 1843.7  [960.2, 3153.2] | 1226.9  [719.1, 2198.9] | 4.3  [3.5, 4.8] | 4.1  [3.6, 4.5] |
| **Complement activation** |  | | | |
| C3bc (nmol/L) | 84.9  [64.8, 115.5] | 71.2  [61.8, 91.0] | 45.5  [6.6, 69.6] | 27.2 [19.0, 72.5] |
| C4bc (nmol/L) | 296.5  [204.2, 344.4] | 143.0  [114.0, 195.8] | 3.5  [0.6, 9.0] | 8.5  [3.1, 17.3] |
| MBL (µg/mL) | 4.3  [2.2, 6.0] | 3.2  [2.2, 4.0] | 0.0  [0.0, 0.0] | 0.0  [0.0, 0.0] |
| **Cytokine release** |  | | | |
| TNF-α | 14.8  [9.3, 22.1] | 16.8  [12.8, 24.6] | 53.6  [13.6, 62.7] | 18.2  [13.6, 67.3] |
| IL-1α | 32.4  [22.1, 48.4] | 20.2  [17.6, 27.0] | 28.3  [9.5, 100.1] | 13.1  [8.9, 16.1] |
| IL-1ß | 7.7  [3.2, 11.5] | 2.8  [2.8, 4.5] | 705.7  [38.1, 4126.2] | 68.7  [14.8, 212.2] |
| IL-1RA | 4835.8  [3142.8, 7288.6] | 6145.3  [2890.8, 8697.9] | 17941.1  [6688.0, 20804.1] | 7040.6  [6010.0, 11786.5] |
| IL-6 | 67.8  [57.3, 98.1] | 36.1*  [12.0, 51.9] | 1290.8  [35.0, 3115.2] | 254.0  [110.3, 503.4] |
| IL-10 | 460.4  [379.3, 622.1] | 345.9  [297.1, 565.9] | 2649.5  [2132.9, 3533.8] | 6356.4 *  [3996.3, 7926.8] |
| IL-33 | 15.0  [12.3, 26.3] | 14.3  [8.9, 14.3] | 22.6  [10.3, 38.8] | 28.6  [13.1, 65.5] |
| **Chemokine release** |  | | | |
| CXCL1 (GROα) | 45.0  [32.3, 76.6] | 67.6  [41.2, 120.4] | 12364.3  [6906.2, 25790.5] | 14924.6  [4573.1, 25644.1] |
| CXCL2 (GROß) | 235.2  [105.4, 318.9] | 449.2  [314.3, 583.0] | 990.0  [267.2, 3011.1] | 381.3  [218.8, 1122.5] |
| CXCL8 (IL-8) | 11.8  [10.3, 12.6] | 9.1  [3.6, 29.2] | 43906.0  [13631.2, 43906.0] | 11351.1  [3056.7, 43906.0] |
| CX3CL (Fractalkine) | 606.8  [540.2, 759.5] | 732.3  [500.7, 860.2] | 571.5  [216.5, 692.7] | 351.4  [254.5, 505.8] |
| CCL3 (MIP-1α) | 20.2  [15.4, 23.9] | 20.2  [18.5, 22.1] | 133.7  [41.5, 408.6] | 90.0  [47.6, 314.7] |
| CCL4 (MIP-1ß) | 186.7  [144.0, 235.5] | 163.6  [129.0, 207.6] | 891.4  [269.8, 1679.5] | 436.4  [368.5, 907.1] |
| CCL5 (RANTES) | 52746.4  [32381.8, 93369.9] | 39041.9  [35539.9, 61111.1] | 47.5  [11.9, 92.0] | 11.9  [11.9, 179.2] |
| CCL18 (PARC) | 274249.3  [186735.9, 424185.2] | 149209.2  [97515.5, 297820.8] | 4762.6  [3175.8, 30076.9] | 30698.6  [8531.4, 40188.0] |
| CCL19 (MIP-3ß) | 559.9  [393.9, 697.7] | 421.5  [308.9, 459.4] | 99.1  [23.9, 154.5] | 50.8  [34.9, 119.4] |
| CCL20 (MIP-3α) | 151.6  [76.6, 237.6] | 137.0  [73.3, 566.1] | 1849.9  [272.7, 3939.0] | 456.2  [259.8, 634.8] |
| **Growth factor release** |  | | | |
| VEGF | 423.2  [348.8, 582.2] | 376.9  [278.7, 560.0] | 501.4  [107.5, 1136.2] | 215.5  [131.3, 345.7] |
| PDGF-AA | 7653.6  [3951.0, 9280.6] | 1717.8  [1454.4, 3501.6] | 76.4  [21.5, 130.9] | 65.9  [23.0, 105.1] |
| PDGF-BB | 2022.9  [1231.2, 3485.8] | 706.6  [494.2, 1257.5] | 4.8  [2.6, 7.0] | 6.4  [5.0, 9.1] |
| FTL3L | 180.4  [170.9, 213.0] | 177.2  [150.1, 209.1] | 596.6  [306.0, 832.7] | 497.8  [335.0, 685.4] |
| GM-CSF | 136.2  [103.9, 194.4] | 91.6  [72.3, 184.0] | 63.1  [48.2, 101.1] | 247.5 **  [130.5, 357.4] |

Data are expressed as medians with the interquartile range between brackets. Significance was tested using a Wilcoxon rank sum test. * p ≤ 0.05, ** p ≤ 0.01. C1-INH = C1-inhibitor; Kallikrein-C1-INH = Kallikrein-C1-inhibitor complexes; PAI-1 = plasminogen activator inhibitor type I; sCD40L = soluble CD40 Ligand; sP-selectin = soluble P-selectin; TATc = thrombin-antithrombin complexes; tPA = tissue type plasminogen activator; C3bc = complement 3bc; C4bc = complement 4bc; MBL = mannose binding lectin; IL = interleukin; IL-1RA = inerleukin-1 receptor antagonist; TNF-α = tumor necrosis factor-α; MIP = macrophage inflammatory protein; PARC = pulmonary and activation-regulated chemokine; RANTES = Regulated upon Activation, Normal T Cell Expressed and Presumably Secreted; FTL3L = fms like tyrosine kinase 3 ligand; GM-CSF = granulocyte-macrophage colony-stimulating factor; PDGF = platelet derived growth factor; VEGF = vascular endothelial growth factor.


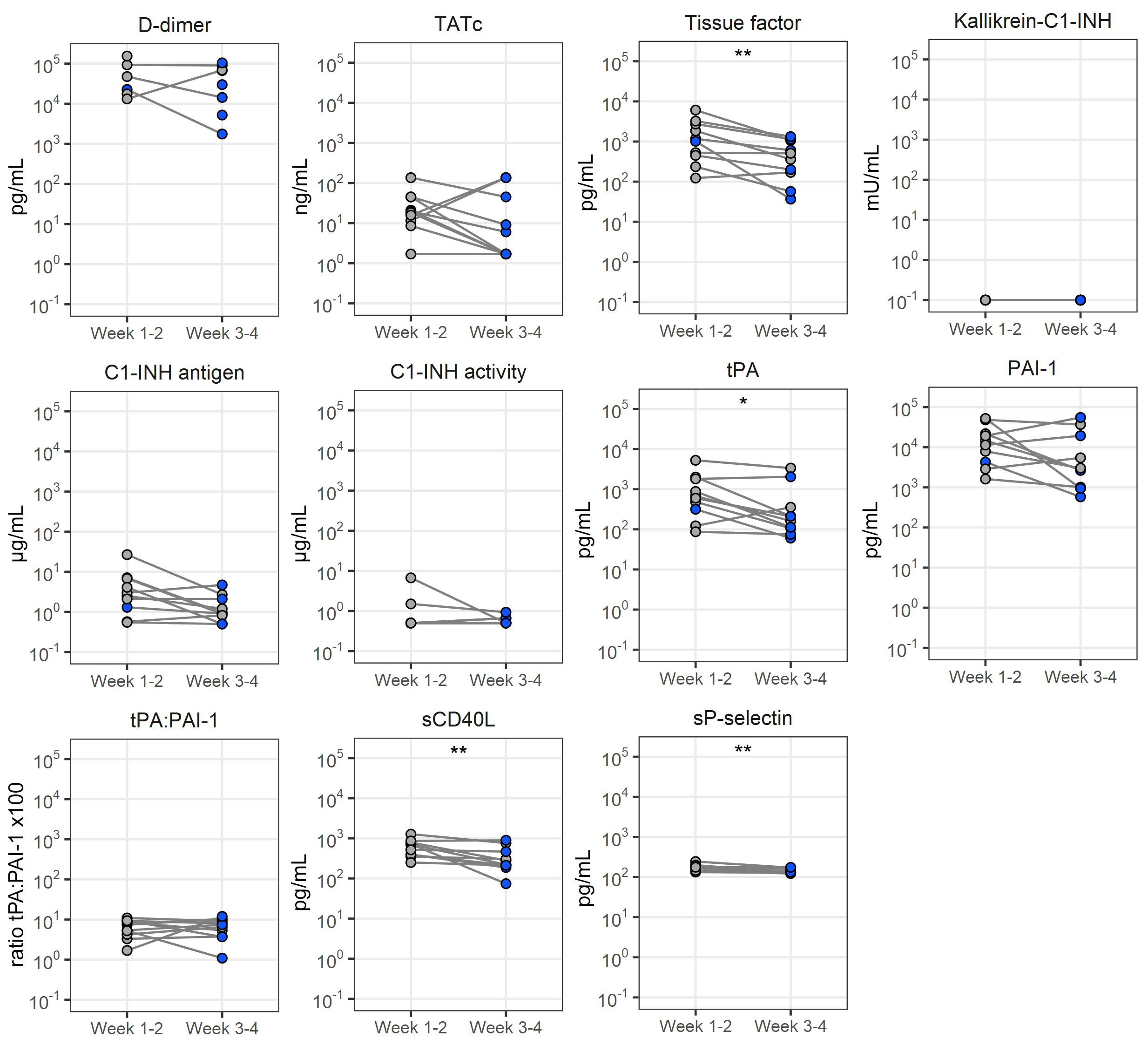


**Supplementary Figure E1. Temporal changes coagulation activation bronchoalveolar space** Biomarker levels reflecting coagulation activation in bronchoalveolar lavage fluid from COVID-19 patients obtained 1-2 weeks and 3-4 weeks after ICU admission. Lines connect paired data from individual patients. Comparisons between time points were performed using the Wilcoxon signed-rank test. * p ≤ 0.05, ** p ≤ 0.01. C1-INH = C1-inhibitor; Kallikrein-C1-INH = Kallikrein-C1-inhibitor complexes; PAI-1 = plasminogen activator inhibitor type I; sCD40L = soluble CD40 Ligand; sP-selectin = soluble P-selectin; TATc = thrombin-antithrombin complexes; tPA = tissue type plasminogen activator. Grey and blue symbols represent samples obtained before or after initiation of steroid treatment respectively.

**
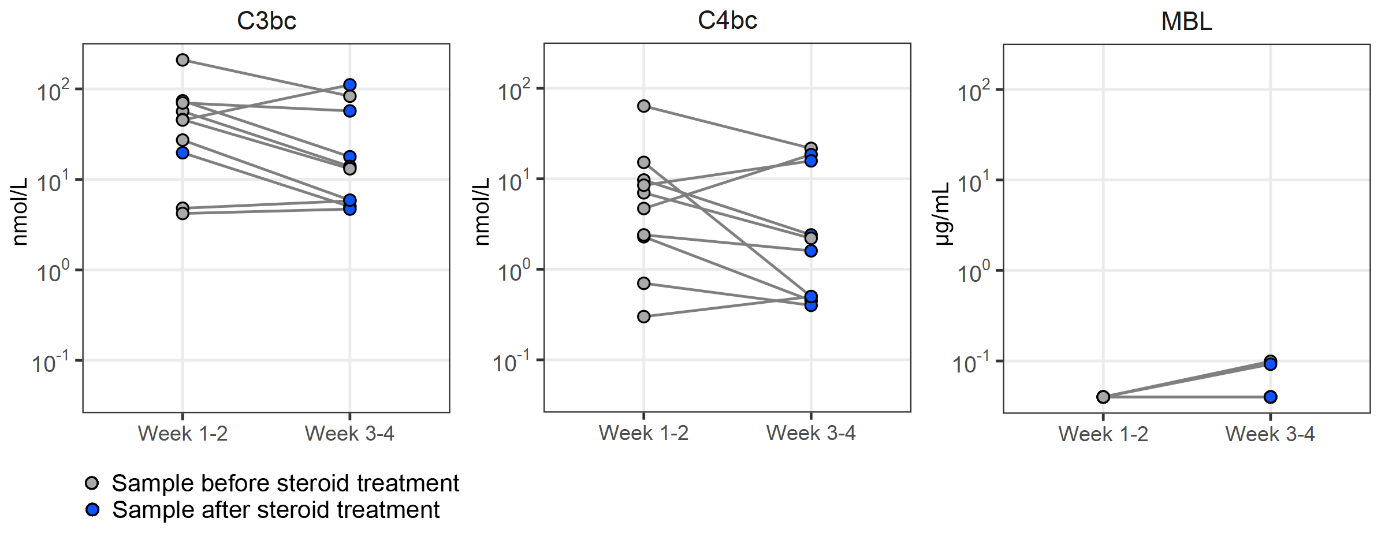
**

**Supplementary Figure E2. Temporal changes complement activation bronchoalveolar space** Biomarker levels reflecting complement activation in bronchoalveolar lavage fluid from COVID-19 patients obtained 1-2 weeks and 3-4 weeks after ICU admission. Lines connect paired data from individual patients. Comparisons between time points were performed using the Wilcoxon signed-rank test. C3bc = complement 3bc; C4bc = complement 4bc; MBL = mannose binding lectin. Grey and blue symbols represent samples obtained before or after initiation of steroid treatment respectively.

**
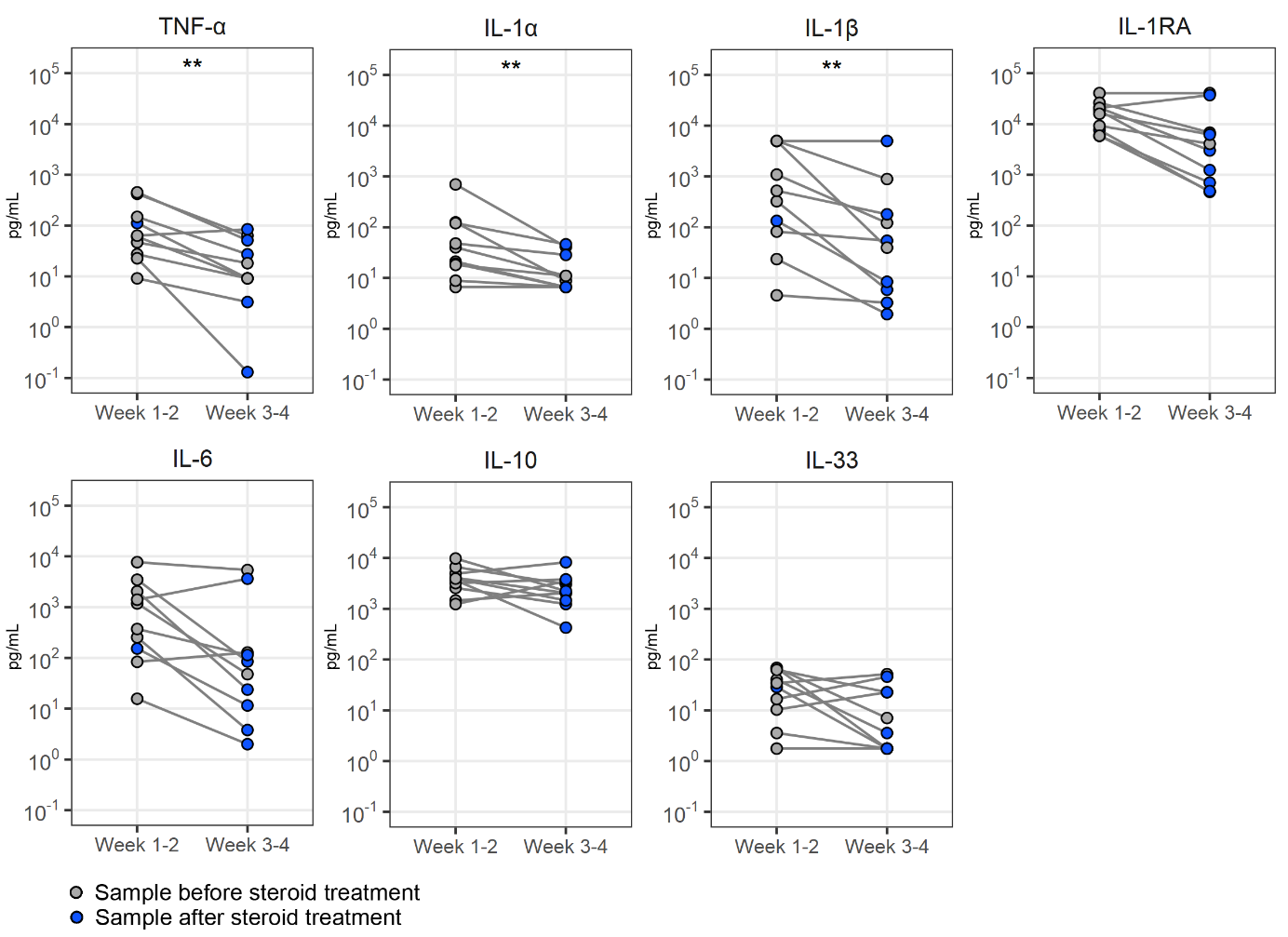
**

**Supplementary Figure E3. Temporal changes cytokine release bronchoalveolar space** Cytokine levels in bronchoalveolar lavage fluid from COVID-19 patients obtained 1-2 weeks and 3-4 weeks after ICU admission. Lines connect paired data from individual patients. Comparisons between time points were performed using the Wilcoxon signed-rank test. ** p ≤ 0.01. IL = interleukin; IL-1RA = inerleukin-1 receptor antagonist; TNF-α = tumor necrosis factor-α. Grey and blue symbols represent samples obtained before or after initiation of steroid treatment respectively.

**
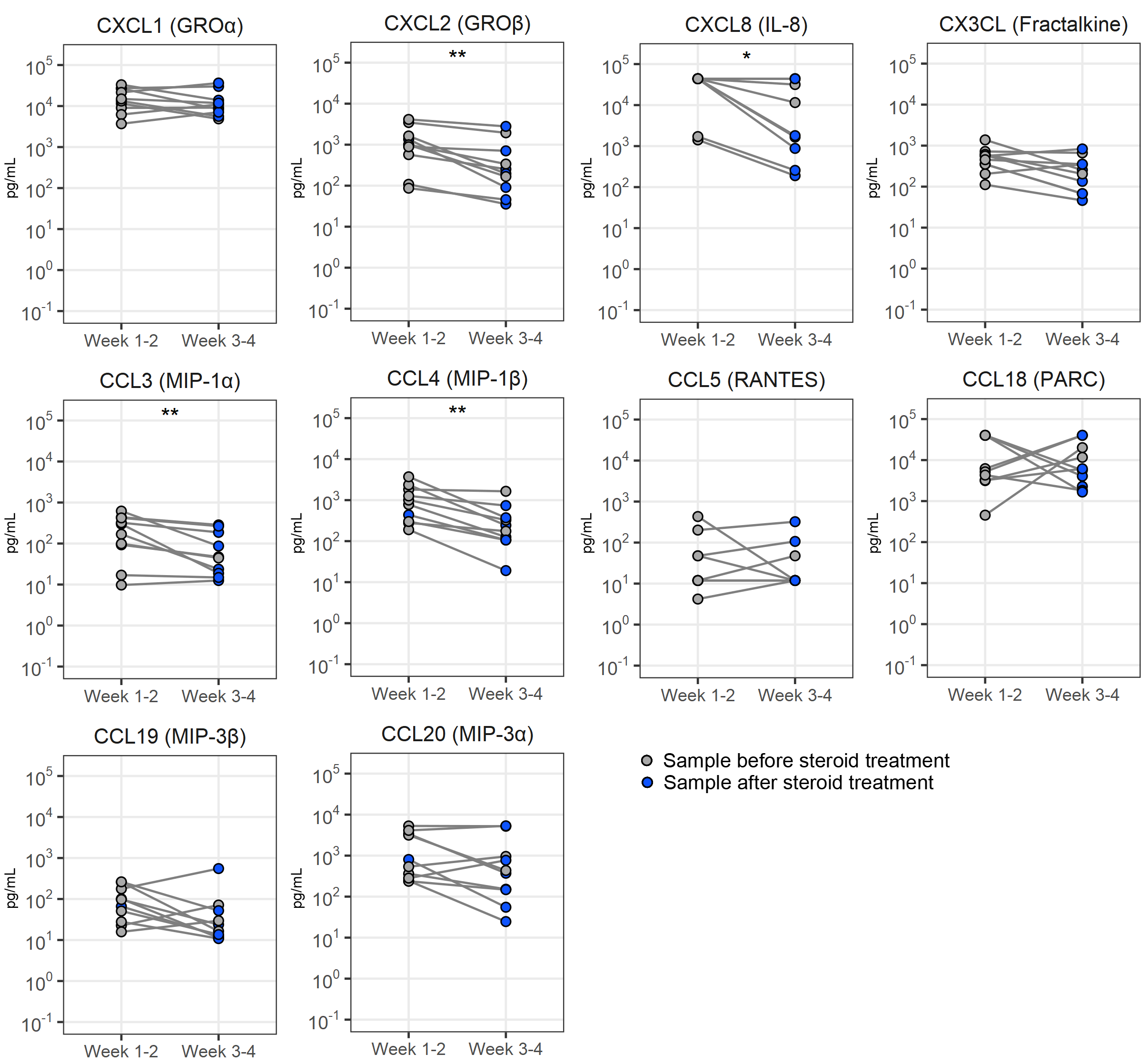
**

**Supplementary Figure E4. Temporal changes chemokine release bronchoalveolar space** Chemokine levels in bronchoalveolar lavage fluid from COVID-19 patients obtained 1-2 weeks and 3-4 weeks after ICU admission. Lines connect paired data from individual patients. Comparisons between time points were performed using the Wilcoxon signed-rank test. * p ≤ 0.05, ** p ≤ 0.01. IL = interleukin; MIP = macrophage inflammatory protein; PARC = pulmonary and activation-regulated chemokine; RANTES = Regulated upon Activation, Normal T Cell Expressed and Presumably Secreted. Grey and blue symbols represent samples obtained before or after initiation of steroid treatment respectively.

**
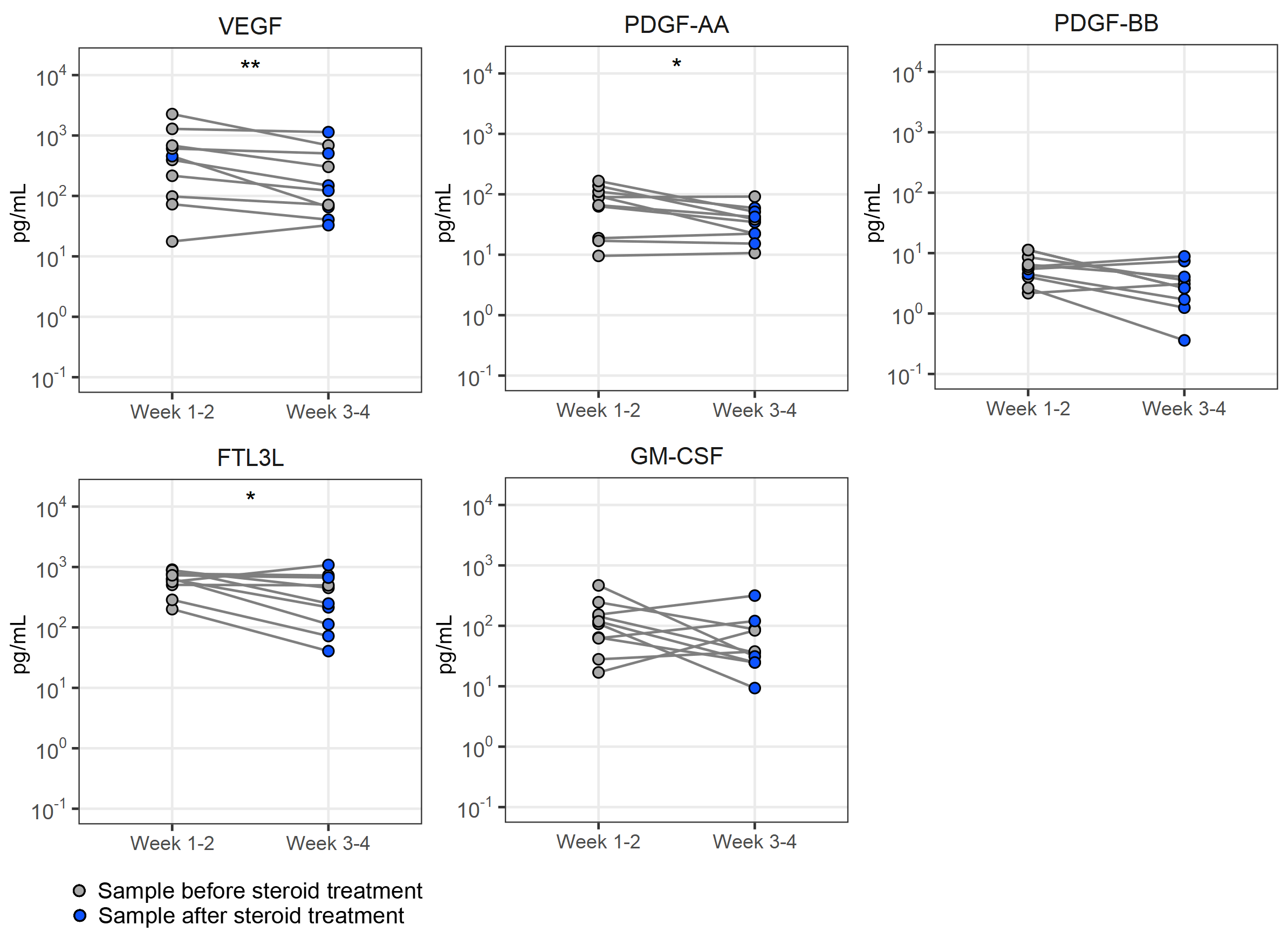
**

**Supplementary Figure E5. Temporal changes growth factor release bronchoalveolar space** Growth factor levels in bronchoalveolar lavage fluid from COVID-19 patients obtained 1-2 weeks and 3-4 weeks after ICU admission. Lines connect paired data from individual patients. Comparisons between time points were performed using the Wilcoxon signed-rank test. * p ≤ 0.05, ** p ≤ 0.01. FTL3L = fms like tyrosine kinase 3 ligand; GM-CSF = granulocyte-macrophage colony-stimulating factor; PDGF = platelet derived growth factor; VEGF = vascular endothelial growth factor. Grey and blue symbols represent samples obtained before or after initiation of steroid treatment respectively.


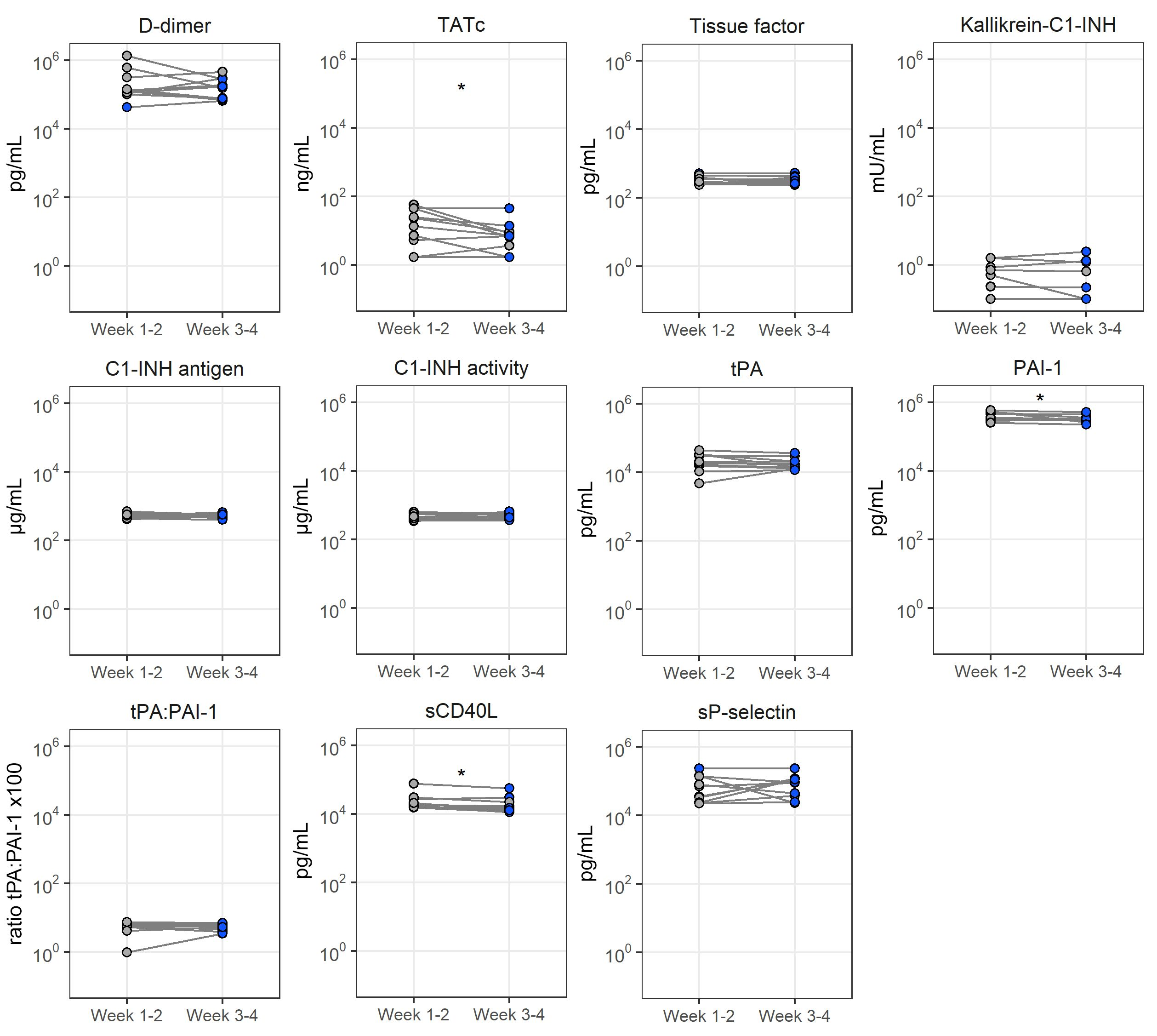


**Supplementary Figure E6. Temporal changes systemic coagulation activation**

Biomarker levels reflecting coagulation activation in plasma from COVID-19 patients obtained 1-2 weeks and 3-4 weeks after ICU admission. Lines connect paired data from individual patients. Comparisons between time points were performed using the Wilcoxon signed-rank test. * P ≤ 0.05. C1-INH = C1-inhibitor ; Kallikrein-C1-INH = Kallikrein-C1-inhibitor complexes; PAI-1 = plasminogen activator inhibitor type I; sCD40L = soluble CD40 Ligand; sP-selectin = soluble P-selectin; TATc = thrombin-antithrombin complexes; tPA = tissue type plasminogen activator. Grey and blue symbols represent samples obtained before or after initiation of steroid treatment respectively.

**
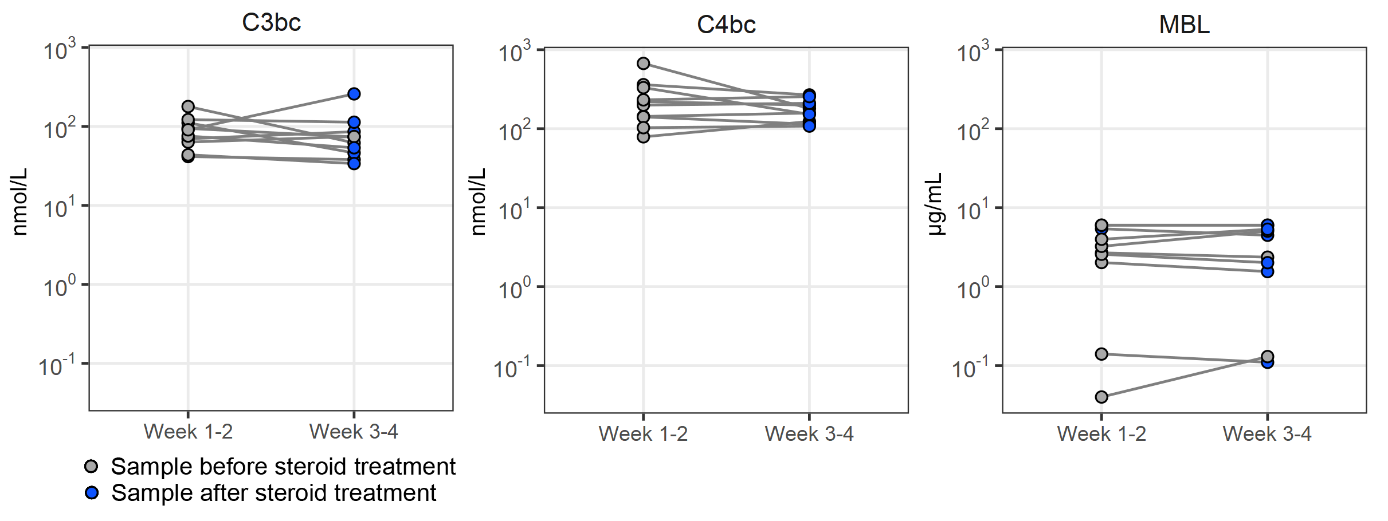
**

**Supplementary Figure E7. Temporal changes systemic complement activation**

Biomarker levels reflecting complement activation in plasma from COVID-19 patients obtained 1-2 weeks and 3-4 weeks after ICU admission. Lines connect paired data from individual patients. Comparisons between time points were performed using the Wilcoxon signed-rank test. C3bc = complement 3bc; C4bc = complement 4bc; MBL = mannose binding lectin. Grey and blue symbols represent samples obtained before or after initiation of steroid steroid treatment respectively.


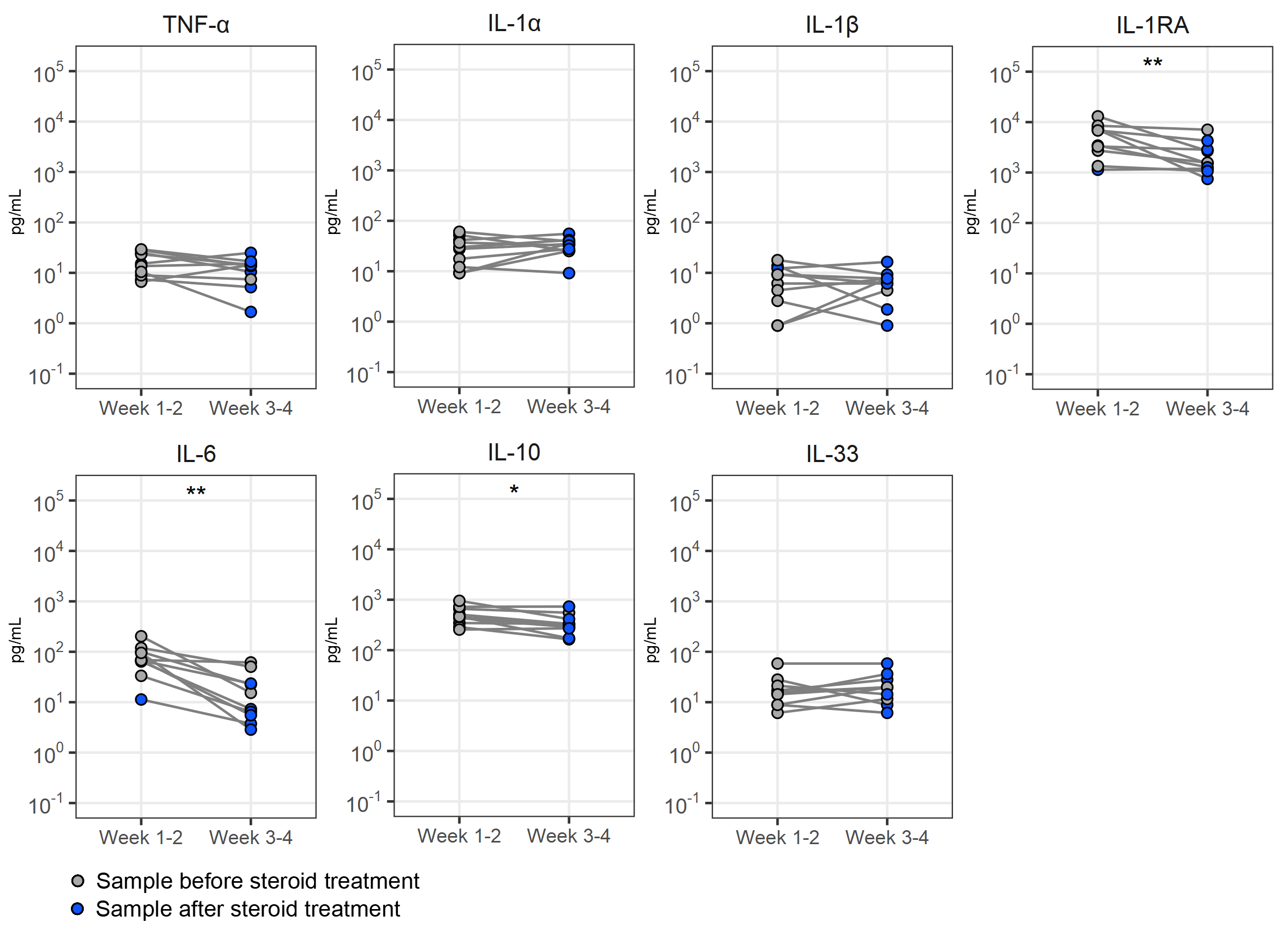


**Supplementary Figure E8. Temporal changes systemic cytokine release**

Cytokine levels in plasma from COVID-19 patients obtained 1-2 weeks and 3-4 weeks after ICU admission. Lines connect paired data from individual patients. Comparisons between time points were performed using the Wilcoxon signed-rank test. * p ≤ 0.05, ** p ≤ 0.01. IL = interleukin; IL-1RA = inerleukin-1 receptor antagonist; TNF-α = tumor necrosis factor-α. Grey and blue symbols represent samples obtained before or after initiation of steroid treatment respectively.

**
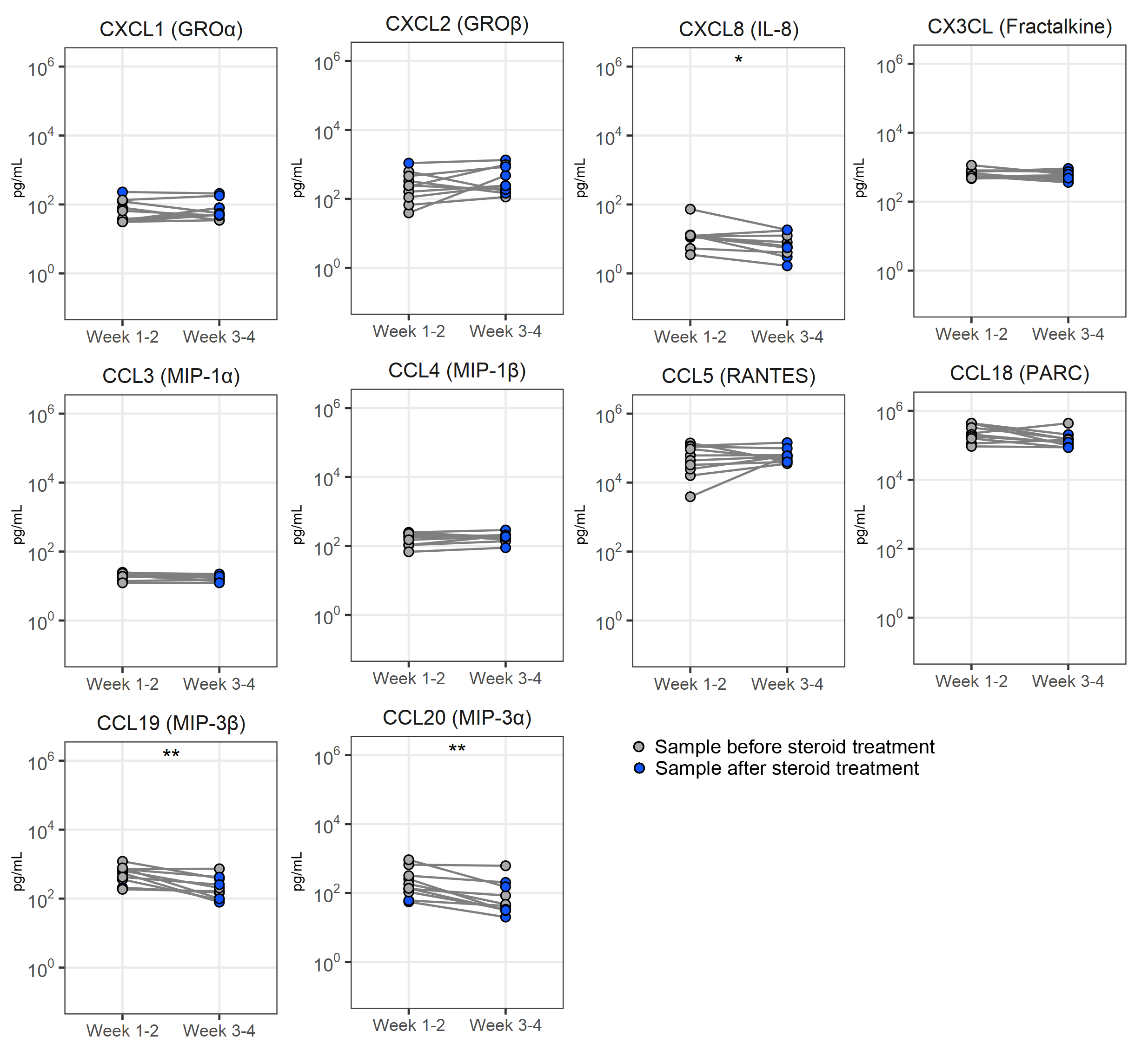
**

**Supplementary Figure E9. Temporal changes systemic chemokine release**

Chemokine levels in plasma from COVID-19 patients obtained 1-2 weeks and 3-4 weeks after ICU admission. Lines connect paired data from individual patients. Comparisons between time points were performed using the Wilcoxon signed-rank test. * p ≤ 0.05, ** p ≤ 0.01. IL = interleukin; MIP = macrophage inflammatory protein; PARC = pulmonary and activation-regulated chemokine; RANTES = Regulated upon Activation, Normal T Cell Expressed and Presumably Secreted. Grey and blue symbols represent samples obtained before or after initiation of steroid treatment respectively.

**
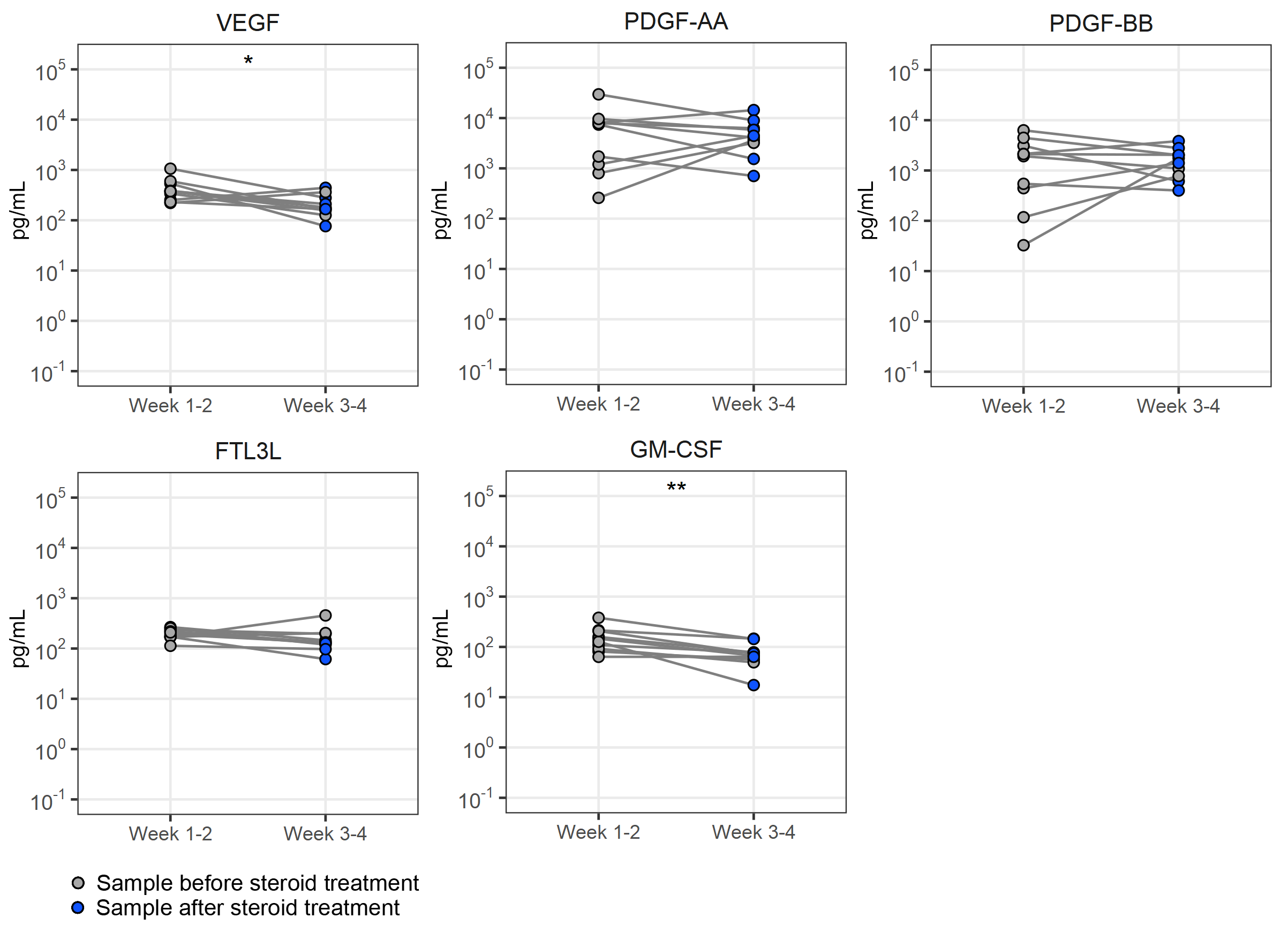
**

**Supplementary Figure E10. Temporal changes systemic growth factor release**

Growth factor levels in plasma from COVID-19 patients obtained 1-2 weeks and 3-4 weeks after ICU admission. Lines connect paired data from individual patients. Comparisons between time points were performed using the Wilcoxon signed-rank test. * p ≤ 0.05, ** p ≤ 0.01. FTL3L = fms like tyrosine kinase 3 ligand; GM-CSF = granulocyte-macrophage colony-stimulating factor; PDGF = platelet derived growth factor; VEGF = vascular endothelial growth factor. Grey and blue symbols represent samples obtained before or after initiation of steroid treatment respectively.
